# Postnatal Immune Activation: A Hidden Trigger for Mood Disorders?

**DOI:** 10.3390/ijms27219757

**Published:** 2026-10-30

**Authors:** Danping Shen, Guanhong Ji, Xinyi Sun, Shubei Ma, Tiantian Zhao

**Affiliations:** 1 Department of Neurology, Central Hospital of Dalian University of Technology, Dalian 116033, China; sweetiee@126.com (D.S.); jiguanhong005@163.com (G.J.); sxy_april@163.com (X.S.); 2 School of Basic Medical Sciences, Faculty of Medicine, Dalian University of Technology, Dalian 116024, China

**Keywords:** postnatal immune activation, neuroinflammation, microglia, astrocyte, mood disorders

## Abstract

Postnatal immune activation (PIA) is linked to persistent CNS homeostatic imbalance, which may contribute to the emergence of neuropsychiatric disorders such as depression and cognitive impairment. This process involves multiple pathological changes such as abnormal activation of central glial cells, neuroimmune disorders, and abnormal neural development during the embryonic period and the early postnatal period. Currently, the specific molecular mechanism by which PIA leads to the occurrence of emotional disorders is still not fully understood. This review summarizes evidence from animal models linking early postnatal immune challenges with later cognitive, anxiety-like, depression-like, and social outcomes, and systematically elaborates on the regulatory role of PIA in the occurrence and development of emotional disorders, aiming to deeply analyze its potential pathological mechanism and provide a basis for early prevention and targeted intervention in mental diseases such as depression and anxiety.

## 1. Introduction

The perinatal period represents a critical developmental window for the structural establishment and functional maturation of the central nervous system (CNS). Adverse perinatal insults, including infection, hypoxia and inflammatory stimuli, can elicit long-term neurodevelopmental reprogramming and substantially elevate susceptibility to neuropsychiatric disorders in adulthood [1,2,3,4,5,6,7]. Postnatal immune activation (PIA) describes systemic immune responses and inflammatory cascades triggered by infectious or infection-mimicking stimuli during the neonatal period which represent a potential risk factor for later cognitive, social and mood-related outcomes in experimental models [1,8,9,10,11]. In addition, some studies have shown that inflammatory markers in childhood can correlate with depressive outcomes in adulthood [12,13]. Current clinical antidepressants predominantly target monoaminergic signaling in adults, yet their clinical utility is constrained by delayed therapeutic onset, partial treatment responses, prominent adverse effects, and limited capacity for disease remission. Furthermore, stringent safety concerns surrounding neonatal pharmacotherapy impose major barriers to early intervention strategies. Although maternal immune activation (MIA) has been extensively characterized, the cellular and molecular mechanisms whereby independent postnatal immune-activating events precipitate neurobehavioral abnormalities remain incompletely systematized. This review summarizes the impacts of PIA on neurobehavioral phenotypes, the central role of glial cells in neuroinflammation, key regulatory signaling cascades, and recent advances in intervention research, with the aim of informing mechanistic insights and therapeutic-preventive frameworks for emotion-related disorders driven by early-life immune stress.

## 2. Postnatal Immune Activation and Offspring Neurobehavioral Outcomes

The neonatal immune system is functionally immature and exhibits heightened sensitivity to inflammatory stimuli; moreover, compared with the MIA reported in the existing research literature, there are significant differences (Table 1). Early postnatal peripheral immune activation increases blood–brain barrier permeability, impairs barrier integrity in the developing brain, initiates central inflammatory cascades, and disrupts neuronal proliferation and differentiation, synaptogenesis and neural circuit maturation; it has also been associated with heterogeneous later outcomes, including cognitive, social, anxiety-like and depression-like measures [12,14]. These outcomes vary with the immune stimulus, exposure window, dose, species or strain, sex, and age at behavioral assessment [15].

### 2.1. Learning and Memory Impairment

Early postnatal infection contributes to cognitive dysfunction in offspring, particularly deficits in learning and memory [16]. A rat study demonstrated that a single episode of inflammatory injury in early postnatal life is sufficient to reshape the developmental trajectory of the neonatal brain [17]. In rodent models, low-dose lipopolysaccharide (LPS) administered in early postnatal life induces interleukin-6 (IL-6) accumulation during the juvenile phase; secondary LPS challenge in adulthood subsequently provokes robust cognitive and behavioral aberrations [12,16].

### 2.2. PIA and Anxiety-like Behaviors

Neonatal inflammatory perturbation disrupts organismal homeostasis, and drives lifelong modifications in brain function, endocrine output and behavioral repertoires. Anxiety-like outcomes after PIA are less consistent across rodent studies than social or depression-like measures, and can be sex-dependent [15]. Altered hypothalamic–pituitary–adrenal (HPA) axis activity and glucocorticoid responses are plausible contributors, but their involvement should be evaluated within each model. Pathological upregulation of pro-inflammatory mediators such as tumor necrosis factor-α (TNF-α) and IL-1β during early ontogenesis directly remodels anxiety-relevant neural circuits and facilitates the emergence of anxiety-related pathologies [18,19,20].

### 2.3. PIA and Depressive-like Behaviors

Population-based cohort studies show that early-life inflammatory exposure correlates with elevated risks of depression and psychosis in adulthood. Prospective human cohorts associate inflammatory markers measured in later childhood, particularly IL-6 at age 9, with subsequent depressive or psychotic outcomes; these findings do not directly test neonatal PIA [13,20]. In rodent PIA models, depressive-like measures represent one component of a broader behavioral phenotype rather than a specific or inevitable consequence. A systematic review published between 2015 and 2025 reported social deficits in 83% of the included studies, and depression-like phenotypes in 73%, while anxiety-related outcomes were more variable. These percentages describe study-level findings, not the proportion of affected animals. Tail-suspension, forced-swim and sucrose-preference outcomes should therefore be interpreted as assay-specific measures, alongside social, cognitive and anxiety-related tests, rather than as evidence that PIA reproduces clinical depression [15].

### 2.4. PIA and Social Behavior

Social behavior is another frequently reported domain after neonatal immune challenge. In mice exposed to LPS on postnatal day 4, reduced adult sociability, social interaction and social memory were observed in females but not in males [21]. Repeated neonatal poly(I:C) exposure or neonatal murine cytomegalovirus infection was associated with adolescent social deficits, predominantly in males in a different experimental setting [22]. The opposing sex patterns across these paradigms should not be treated as a pooled sex effect; they instead illustrate dependence on the immune challenge, developmental window and assay [15,21,22].

## 3. Animal Models

Experimental models of postnatal immune activation (PIA) differ in the immune stimulus, the route and timing of exposure, and the extent to which they approximate infection in a developing organism (Table 2). Accordingly, model choice should be linked to the biological question rather than to the expectation of a uniform mood-related outcome.

Neonatal LPS administration is an experimentally tractable bacterial-mimetic paradigm that primarily engages Toll-like receptor 4 (TLR4)-associated innate immune signaling. It permits controlled manipulation of postnatal age, dose and route, and has been used to examine later behavioral and neuroimmune outcomes [2,7,23,24,25,26]. Its construct validity is therefore strongest for selected consequences of exposure to a Gram-negative bacterial component, not for neonatal infection as a whole. Although some studies report later depression-like measures, an isolated LPS challenge does not reproduce pathogen replication, the full host response, or the clinical syndrome of depression. LPS dose, strain, sex, developmental stage and the timing of testing can change the observed phenotype [15]. Postnatal polyinosinic:polycytidylic acid [poly(I:C)] exposure provides a complementary viral-mimetic approach through double-stranded RNA sensing, including TLR3-associated signaling. Poly(I:C) administered to rat pups at postnatal day 7 was associated with later spatial and fear-memory deficits [27]. In a more recent mouse study, repeated neonatal poly(I:C) exposure, as well as neonatal murine cytomegalovirus infection, was followed by adolescent social and cognitive deficits, predominantly in males [22]. These findings show that viral-mimetic challenges can produce persistent outcomes, but they do not establish that poly(I:C) and LPS induce the same immune pathways or the same mood-related phenotype.

Live-pathogen models capture pathogen–host interactions that purified immune agonists cannot reproduce. For example, neonatal Escherichia coli infection in mice produced transient brain inflammation followed by later motor abnormalities and hypomyelination [28]. Such models improve biological resemblance to infection but add variation arising from pathogen burden, tissue distribution, illness severity and survival. A separate, indirect postnatal paradigm activates immunity in lactating dams rather than in pups; maternal poly(I:C) exposure altered cytokines in milk and offspring brain and yielded sex-dependent adult behavioral outcomes [29]. This lactational model is informative about maternal–offspring transmission, but it should not be treated as equivalent to direct immune activation of the neonate.

The face validity of any PIA model is limited to the outcomes actually measured, such as cytokine responses, glial changes or specific behavioral tests. Construct validity depends on whether the chosen stimulus reflects the hypothesized immune pathway, while predictive validity requires evidence that an intervention has comparable effects across relevant settings. No single paradigm currently captures the full range of human postnatal infection or later neuropsychiatric outcomes. Cross-model comparisons using matched developmental windows, both sexes, and multiple behavioral domains are needed to determine which findings generalize beyond a particular stimulus [15,22].

## 4. Mechanisms Underlying PIA-Mediated Emotional Disorders

### 4.1. Postnatal Immune Activation and Glial Cells

Cumulative experimental evidence indicates that PIA-induced mood disorders in offspring are tightly coupled with glial activation [30,31,32,33,34,35]. PIA typically manifests during the early postnatal period, when neonates possess immature immune defense and are highly susceptible to infection. Unresolved neonatal infection increases lifelong risk of neuropsychiatric disorders. Pharmacological suppression of astrocytic and microglial activation markedly ameliorates abnormal behaviors [7,33,34]. The cellular focus now shifts to microglia and astrocytes. These cells can respond to inflammatory signals, and influence synaptic maintenance, neurotransmitter handling and trophic support, thereby connecting the pathways summarized in Section 4 with neural circuit outcomes. Microglial involvement has direct support in selected PIA experiments, including an IL-27 intervention that altered microglial synaptic engulfment and later behavior [7]; comparable causal evidence for astrocytic mediation is less developed [15]. The sequence and necessity of each cellular response remain model-dependent questions. The CNS comprises heterogeneous cellular populations encompassing neurons and glial cells. Glia constitute approximately half of all CNS cells, and perform indispensable functions governing neural development and tissue homeostasis. The principal glial subtypes are astrocytes and microglia. Astrocytes originate from CNS-resident neural stem cells; microglia are tissue-resident brain macrophages derived from yolk-sac myeloid progenitors that colonize the CNS at embryonic day 9.5 (E9.5). The functional contributions of astrocytes and microglia across neuropsychiatric pathologies have attracted escalating research attention.

#### 4.1.1. Microglia

Microglia provide a plausible cellular route from neonatal immune challenge to altered synaptic development, although the direction and persistence of their responses vary by model [7,9,15]. In direct PIA models, altered microglial engulfment of neuronal structures has been linked to later depression-like measures; microglial IL-27 manipulation in one neonatal LPS model provides intervention evidence for a microglia–synapse pathway [7,9]. Findings requiring an additional adult stress exposure should be identified as double-hit evidence rather than attributed to PIA alone [24]. Microglia are indispensable for physiological brain development. During early ontogenesis, microglia secrete trophic factors that support neuronal circuit assembly and neuronal survival. For instance, microglia-derived insulin-like growth factor-1 promotes survival of layer V cortical neurons across postnatal development [36]. Approximately half of immature neurons undergo physiological programmed cell death during brain maturation. In the developing mouse hippocampus, microglia facilitate neuronal programmed cell death by releasing reactive oxygen species (ROS) in a CD11b- and DNAX-activating protein 12 (DAP12)-dependent fashion [37]. Beyond apoptotic cell clearance, microglial phagocytosis underpins synaptic homeostasis: microglia execute developmental synaptic pruning, and dynamically respond to synaptic activity and plasticity [38]. Microglia are exquisitely sensitive to extrinsic stressors, rapidly shifting activation states and functional profiles upon challenge. These developmental and disease-model studies provide biological context for microglial functions [36,37,38,39,40,41,42,43]. They do not establish that all microglial state changes after neonatal PIA are harmful, or that suppressing microglia broadly would improve every later behavioral outcome. Cell-specific perturbation and longitudinal phenotyping are needed to identify which microglial responses are necessary in each PIA model [7,15]. Studies of adult stress or acute LPS paradigms suggest that modifying glial responses can alter depressive-like assays [44,45,46,47], but these data do not demonstrate efficacy against later PIA-associated outcomes or a common clinical antidepressant mechanism.

#### 4.1.2. Astrocytes

Astrocytes represent a second cellular interface through which immune signals could affect neuronal metabolism and synaptic function; the PIA-specific evidence for persistent astrocytic mediation remains more limited than that for microglia [15]. As the most abundant cellular population within the brain, astrocytes execute diverse fundamental functions supporting neuronal metabolism and actively modulating neural circuit performance and behavior. Through intercellular contacts, astrocytes participate in cerebral blood-flow regulation, neurotransmitter handling, energy metabolism, signal transduction, ionic buffering and neuronal trophic support. Astrocytic perivascular end-feet form intimate contacts with cerebral vasculature to assemble neurovascular units that govern cerebral blood flow (CBF) and BBB integrity [47]. Astrocytes are key regulators of neuronal energy homeostasis. In the healthy CNS, the majority of synapses are ensheathed by astrocytic processes, which act as signaling intermediaries, energy suppliers and modulators at synaptic junctions. Astrocytes additionally serve as innate immune effector cells within the CNS. Innate immunity orchestrates primary tissue responses to infection or injury, characterized by local secretion of inflammatory mediators (cytokines, chemokines, prostaglandins) that mediate chemotaxis, inflammatory cell recruitment and phagocytic clearance of cellular debris or pathogens. While microglia are widely regarded as primary drivers of CNS innate immune responses, astrocytes can also initiate local innate immune signaling upon diverse stimuli or brain injury via pattern-recognition receptor expression; their phenotypic state is profoundly modulated by pro-inflammatory cytokines. Evidence from adult depression, acute LPS or other neurological models can motivate astrocyte hypotheses but is not direct evidence of neonatal PIA mediation [46,48,49,50]. A direct neonatal LPS study observed astrocytic activation during the second postnatal week together with later neurogenic, GABAergic and depression-like changes [2]. However, its TLR4 intervention was not astrocyte-specific, so it could not isolate astrocytes as the necessary mediator. In one systematic review published in 2026, increased astrocytic GFAP was reported in 29% of included studies and astrocyte morphology was rarely quantified, whereas microglial marker changes were reported in most studies [15]. Unpublished preliminary observations should be presented with methods and data before they are used to support a persistent astrocyte mechanism. Astrocytic glutamate uptake is one plausible interface between inflammation and neuronal excitability. Evidence from broader depression and neuroinflammation models links impaired glutamate clearance with excessive extracellular glutamate and NMDA-receptor stimulation [51,52]; whether the same sequence mediates later behavior after neonatal PIA requires direct testing. Broader depression literature discusses impaired astrocytic glutamate handling as a possible link to neuronal excitability [53], but direct tests of this sequence after neonatal PIA remain lacking.

### 4.2. Regulatory Mechanisms Underlying Glial Activation in PIA

**Figure 1:**
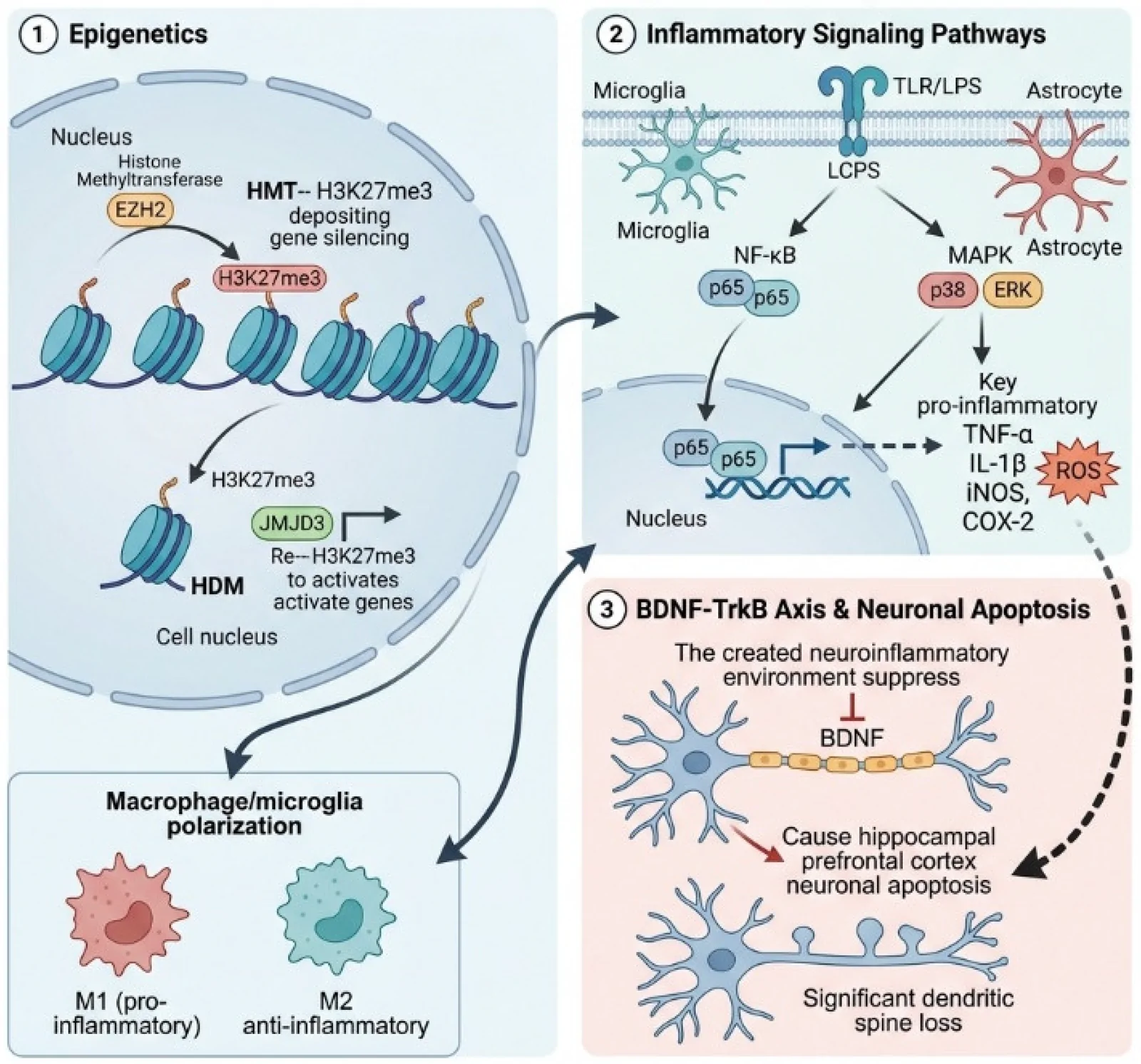
The possible mechanisms related to glial cells in postnatal immune activation.

Epigenetic marks and NF-κB/MAPK signaling are considered candidate regulators of glial state, whereas BDNF-TrkB is considered a possible glia–neuron feedback pathway, rather than a separate upstream cause (Figure 1) [54,55]. Determining which links are upstream, downstream or bidirectional after PIA requires model-specific temporal and intervention data.

#### 4.2.1. Epigenetic Regulatory Mechanisms

Epigenetic regulation can alter gene expression without changing the DNA sequence; its mechanisms include DNA methylation, histone modifications, chromatin organization and non-coding RNA-mediated regulation [56]. Persistence across cell divisions or developmental stages must be demonstrated for a specific mark rather than assumed from this definition. Histone modification constitutes a core epigenetic mechanism whereby amino-acid residues located on the N-terminal tails of histones undergo covalent modification by nuclear enzymatic complexes. Key histone modifications include methylation, acetylation, phosphorylation and ubiquitination [57]. Histone acetylation generally facilitates transcriptional activation, whereas histone methylation exerts context-dependent regulatory effects. Lysine methylation involves addition of one, two or three methyl moieties to the ε-amino group of lysine side-chains, yielding mono-, di- and trimethylated histone variants. Well-characterized histone methylation marks include H3K27me3, H3K9me3 and H3K4me3; H3K27me3 and H3K9me3 predominantly mediate transcriptional repression, whereas H3K4me3 promotes transcriptional initiation [58].

Histone methylation status is dynamically governed by histone methyltransferases (HMTs) and histone demethylases (HDMs), which catalyze methyl-group deposition and removal respectively [59]. Some histone marks can persist after an early-life challenge, but their stability depends on the locus, the cell type and the sampling interval; neonatal PIA-specific longitudinal measurements are needed. Enhancer of zeste homologue 2 (EZH2), a canonical HMT functioning via its SET domain, can either repress or activate gene transcription in a context-dependent manner. EZH2 binds target gene promoters and deposits repressive H3K27me3 marks, leading to transcriptional silencing. Histone methylation modulates inflammatory responses across multiple cell types, including microglia, astrocytes and macrophages; pharmacological inhibition of EZH2 in microglia attenuates LPS-provoked inflammatory signaling [60]. In chronic unpredictable mild stress (CUMS) depression models, EZH2 inhibition upregulates SOCS3 expression and downregulates phosphorylated-NF-κB, TNF-α and IL-1β in the prefrontal cortex and hippocampus, thereby suppressing neuroinflammation and ameliorating depressive-like behaviors [61]. Genetic ablation of Ezh2 accelerates astrocyte generation and differentiation [62]. Macrophage-polarization studies show that inflammatory gene programs can be regulated by histone methylation and acetylation [63,64,65,66]. These peripheral or non-PIA findings cannot identify persistent chromatin states in neonatal PIA-exposed brain microglia. Jumonji domain-containing protein 3 (JMJD3), an H3K27me3 demethylase, is transcriptionally induced downstream of NF-κB and modulates pro-inflammatory cytokine production to shape neuroinflammatory outputs. JMJD3 exacerbates neuroinflammation in the prefrontal cortex and hippocampus and increases vulnerability to depression induced by maternal separation in male rats [67].

In a direct neonatal viral-mimic model, poly(I:C) given on postnatal days 2–6 was followed at day 40 by altered H3K4me3 and H3K9ac at selected promoters in isolated male microglia, together with synaptic loss and social or cognitive deficits; neutralizing IFN-γ during the challenge reduced these molecular and behavioral changes [22]. The marks were demonstrated into adolescence, not adulthood, and the tested outcomes were not depression-like behavior. Because IFN-γ blockade affects several processes, this result does not by itself prove that the histone marks mediate behavior. A neonatal LPS study (postnatal days 3 and 5) found altered adult hippocampal H4K12 acetylation and c-Fos-promoter occupancy during a spatial-memory task; a histone-deacetylase inhibitor improved the memory outcome [24]. This is direct PIA evidence for an adult, task-evoked acetylation response, but it used hippocampal tissue rather than glia-specific chromatin profiling and did not test depression-like behavior. A newer study reported adult ventral-hippocampal Bag3-promoter hypomethylation after postnatal day 4 LPS, with Bag3 deletion and methyl-donor treatment modifying microglial priming and depression-like susceptibility [68]. Its behavioral phenotype required a later subthreshold chronic stressor, so this is double-hit evidence rather than an effect of neonatal PIA alone; the abstract does not establish cell-specific promoter methylation. In addition, some of our previous research work has initially confirmed that histone methylation modifications, especially H3K27me3 and H3K9me3, play a key role in the glia-inflammation of PIA [33,69]. Thus, postnatal immune challenge has been linked to adolescent microglial histone changes, adult hippocampal histone-acetylation responses during memory testing, and adult Bag3-promoter hypomethylation in a neonatal-LPS-plus-adult-stress model. These findings differ in stimulus, sex, cell specificity, sampling time and behavioral outcome. Evidence for a persistent EZH2/JMJD3 pathway, for a PIA-specific non-coding RNA mechanism, or for a chromatin-remodeling complex that mediates depression-like behavior remains insufficient. Future studies should combine time-resolved, cell-specific epigenomic measurements with targeted perturbation and mood-relevant behavioral testing. Together, these studies support epigenetic regulation as a plausible framework for PIA, but they do not yet establish a persistent, cell-specific epigenetic mechanism connecting neonatal PIA to later behavior.

#### 4.2.2. Inflammation-Associated Signaling Pathways

Glial activation and functional remodeling triggered by perinatal-infection-evoked CNS inflammation are precisely coordinated by diverse intracellular inflammatory signaling cascades [70,71,72]. Canonical inflammatory signaling pathways include nuclear factor-κB (NF-κB) and mitogen-activated protein kinase (MAPK) cascades. NF-κB can regulate inflammatory gene expression in glial cells, and MAPK pathways can modulate related responses. However, evidence cited here from intracerebral hemorrhage or other non-PIA settings does not establish that either pathway is necessary for behavioral changes after neonatal PIA [73]. Koo and colleagues first reported that NF-κB signaling acts as a critical mediator of neurogenic- and stress-provoked depressive-like behaviors and represents a promising therapeutic target for depression [74]. Subsequent clinical studies have documented upregulation of redox-sensitive transcription factors Nrf2 and NF-κB in peripheral blood mononuclear cells isolated from MDD patients [75]. A review focusing on adult hippocampal neurogenesis further supports the premise that antidepressant efficacy is partially mediated through NF-κB-dependent modulation of adult neurogenesis [76]. Chronic stress within the basolateral amygdala (BLA) induces neuropeptide upregulation and subsequent depressive-like behaviors; siRNA-mediated suppression of BLA-localized NF-κB signaling downregulates neuropeptide expression and alleviates depressive-like symptomatology [77]. Importantly, previous study has indicated that PIA can induce activation of the NF-κB signaling pathway; conversely, pharmacological inhibition of this pathway effectively alleviates PIA-induced mood disorders [34]. Similarly, several small-molecule compounds exhibit therapeutic potential against LPS-induced depressive-like behaviors and neuroinflammation. Saffron, a natural product endowed with anti-inflammatory and antioxidant properties, suppresses LPS-mediated depressive-like phenotypes by inhibiting microglial NLRP3 inflammasome assembly and NF-κB signal transduction [78]. Grape-seed-derived proanthocyanidins modulate NF-κB signaling across multiple brain regions and repress LPS-induced iNOS and COX-2 expression, marking them as candidate therapeutics for LPS-triggered depressive-like behaviors [79]. Astrocytic dysfunction and astrocyte-propagated inflammation contribute to MDD pathogenesis. The MEN1 gene (encoding Menin protein) is significantly downregulated in brains of LPS-challenged mice exhibiting depressive-like behaviors. Enforced Menin over-expression inhibits phosphorylation of the NF-κB p65 subunit, dampens LPS-stimulated astrocyte-mediated inflammatory responses, and rescues resultant depressive-like behavioral deficits [80]. These adult stress, acute LPS and other non-PIA findings generate testable hypotheses, but neonatal PIA-specific interventions and safety data are required before proposing NF-κB-targeted treatment for later mood outcomes. Glial activation in the setting of CNS inflammation is fine-tuned by multiple intersecting signaling pathways to sustain cerebral immune microenvironment homeostasis. Further characterization of inflammatory signaling mechanisms controlling glial activation will facilitate the discovery of novel glia-targeted antidepressant and anxiolytic pharmacotherapies.

#### 4.2.3. BDNF-TrkB Signaling

Brain-derived neurotrophic factor (BDNF), one of the most prominent cerebral neurotrophic factors, supports neuronal survival, modulates neural plasticity, and shapes neurotransmitter signaling. BDNF is widely recognized as an “antidepressant-like molecule”, and its cerebral expression is frequently diminished in patients with neuropsychiatric and neurodegenerative disorders [81,82,83,84]. This molecular phenotype may stem from neuroinflammatory suppression of BDNF-associated signal transduction. PIA provokes glial activation, which elevates pro-/anti-inflammatory cytokine and ROS abundance and consequently suppresses cerebral BDNF expression [85]. Experimental LPS challenge markedly reduces both BDNF transcript and protein abundance [86]. Reduced BDNF levels amplify neuronal apoptosis and cellular injury, which in turn further potentiates glial activation, establishing a feed-forward pathogenic vicious cycle. Cumulative evidence underscores the indispensable role of BDNF in depression pathophysiology and antidepressant therapeutic mechanisms. Intra-cerebral BDNF infusion elicits robust antidepressant effects in learned-helplessness rat depression models, and these therapeutic benefits are abolished by pan-Trk inhibitor K252a [87]. Post mortem human brain analyses reveal diminished BDNF concentrations within the hippocampus and prefrontal cortex of depressive patients [88]. Collectively, these findings provide a mechanistic rationale for deploying anti-inflammatory agents to ameliorate depressive symptomatology. Previous studies summarized contemporary evidence that BDNF–TrkB signaling participates in activity-dependent plasticity and antidepressant responses [89,90]. The narrower PIA question is whether early inflammatory glial responses alter BDNF–TrkB signaling in defined cell populations and whether subsequent neuronal changes feedback on glia; the cited literature on broader inflammation cannot establish this sequence [84,85,91]. One neonatal LPS study found early astrocytic-marker changes together with reduced adult hippocampal BDNF-TrkB signaling [2], but did not establish an astrocyte-specific causal chain. Broader neuroinflammation and non-neonatal depression studies provide context for but not evidence of a universal glia-BDNF feedback loop after PIA [85,92]. Evidence from learned-helplessness models and human post mortem studies supports the broader relevance of BDNF to depression, but does not by itself identify the direction of glia-BDNF signaling after neonatal PIA [86,87,88]. Cell-specific, time-resolved manipulations are needed to test whether this pathway mediates PIA-associated behavioral changes.

## 5. Advances in Pharmacotherapeutic Research

Safety represents the paramount consideration in neonatal clinical pharmacotherapy. Conventional antidepressants in current clinical practice are developed primarily for adult patients and carry substantial adverse-effect burdens. Given the close pathogenic linkage between depression and PIA, neonatal-targeted pharmacological intervention holds promising translational potential for treating PIA-associated depressive symptomatology.

### 5.1. Minocycline

Minocycline is a broad-spectrum tetracycline-class antibiotic that exerts bacteriostatic activity via interference with tRNA function. Preclinical studies demonstrate that minocycline effectively suppresses hippocampal neuroinflammation and HPA axis hyperactivation, thereby ameliorating PIA-provoked anxiety- and depressive-like behaviors [93].

### 5.2. Fluoxetine

Fluoxetine is a widely prescribed selective serotonin reuptake inhibitor (SSRI) in clinical practice. Its established primary pharmacological action is serotonin transporter inhibition. Beyond this immediate action, experimental studies implicate plasticity-related pathways, including BDNF–TrkB signaling; TrkB-binding findings come from non-PIA systems and should not be assumed to mediate the neonatal intervention results below [89,90]. In two mouse studies, LPS exposure on postnatal days 3 and 5 was followed by fluoxetine treatment during the peri-adolescent period (postnatal days 35–65), not during the neonatal exposure window. One study reported reduced adult depression-like measures [6], whereas the other reported reduced adult anxiety-like measures [93] These model-specific findings do not establish neonatal dosing, uniform prevention, or clinical efficacy; both studies also warrant attention to effects in unchallenged control mice.

## 6. Summary and Future Perspectives

PIA studies show that selected postnatal immune challenges can be followed by lasting neuroinflammatory, glial, developmental, and behavioral changes. However, these effects vary across models, and their relevance to lifelong psychiatric risk in humans remains uncertain [15]. Microglial responses have some direct support from intervention studies, whereas persistent astrocyte-specific effects and causal links among NF-κB/MAPK, epigenetic, and BDNF–TrkB pathways remain less well established in PIA models [7,15]. These pathways should therefore be treated as interacting candidates for further study, not as a single confirmed causal chain. Model choice also limits interpretation: neonatal LPS is a useful bacterial-mimetic paradigm, whereas poly(I:C) and live-pathogen models engage different immune processes and may yield different outcomes [15,22]. Evidence for minocycline and peri-adolescent fluoxetine is preclinical and model-specific [6,93]; it does not yet justify neonatal clinical treatment. Future studies should compare immune challenges across matched developmental windows, doses, sexes, and behavioral domains; establish the temporal and cell-specific relationships among glial, endocrine, synaptic, and epigenetic changes; and test whether targeted interventions alter later outcomes without unacceptable developmental risks. Carefully controlled human cohorts are also needed to determine whether early postnatal immune perturbations independently contribute to later mood disorders. Depression-like measures should be considered alongside social, cognitive, and anxiety-related outcomes, with explicit attention to sex and experimental context [15,21,22].

## Figures and Tables

**Table 1:** Comparison between MIA and PIA.

Category	Maternal Immune Activation (MIA)	Postnatal Immune Activation (PIA)
Timing of immune challenge	Prenatal period, typically mid-to-late gestation in mice (e.g., embryonic day E9–E14). Immune stimulation is applied to pregnant dams before parturition.	Early postnatal neonatal window, usually P3–P14 in mice, corresponding to the brain rapid developmental/growth spurt period. Immune stimulation is administered directly to pups after birth.
Commonly used stimuli	Poly(I:C) (viral mimetic), LPS (bacterial mimetic), recombinant IL-6, or inflammatory cytokines injected into pregnant dams.	LPS, Poly(I:C), pro-inflammatory cytokines, live pathogen or pathogen-associated molecular patterns, directly injected to neonatal pups. Dams receive no immune challenge.
Primary affected brain regions	Prefrontal cortex, hippocampus, amygdala, cerebellum; primarily disrupts prenatal neurogenesis and neuronal migration.	Hippocampus, developing cerebral cortex, corpus callosum, periventricular white matter; mainly impacts gliogenesis, oligodendrocyte maturation and synaptogenesis.
Behavioral phenotypes	Autism-relevant phenotypes: social interaction deficits, repetitive behaviors, increased anxiety, impaired sensory processing; cognitive deficits.	Depression- and schizophrenia -relevant phenotypes: anhedonia, depressive-like behaviors, impaired working memory, altered HPA axis reactivity, prominent long-term microglial priming.
Key cellular and molecular mechanisms	Inflammatory mediators from the dam cross the placenta; disrupt fetal neuronal proliferation and migration; fetal microglia priming; altered neuronal synaptic formation.	Inflammatory mediators directly act on neonatal brain; robust activation of resident microglia and astrocytes; disrupted myelination; altered synaptic pruning in the postnatal critical developmental window.

**Table 2:** Comparison of experimental paradigms used to study postnatal immune activation.

Paradigm	Construct and Immune Pathway	Useful Features and Observed Outcomes	Main Limitations
Neonatal LPS	Bacterial-component mimic; predominantly TLR4-associated innate response	Controlled dose and timing; later behavioral and neuroimmune readouts	No replicating pathogen; incomplete infection physiology; dose, sex and timing matter
Neonatal poly(I:C)	Viral double-stranded-RNA mimic; TLR3-associated signaling	Direct postnatal challenge; memory, social and cognitive outcomes reported	Not a viral infection; endpoints and sex effects cannot be generalized to LPS
Live neonatal infection	Replicating bacterial or viral pathogen with broader host response	Closer resemblance to infection; longer-term motor, social or cognitive effects	Pathogen burden and illness severity vary; infection-specific pathology
Lactational maternal immune activation	Maternal immune stimulus with indirect exposure of pups	Tests maternal–offspring transmission and sex-dependent outcomes	Not direct neonatal PIA; maternal care and milk composition may contribute

## Data Availability

No new data were created or analyzed in this study. Data sharing is not applicable to this article.

## Abbreviations

The following abbreviations are used in this manuscript:

PIA	Postnatal immune activation
MIA	Maternal immune activation
BBB	Blood–brain barrier
LPS	Lipopolysaccharide
BDNF	Brain-derived neurotrophic factor
CNS	Central nervous system
CUMS	Chronic unpredictable mild stress
